# A Location Method Using Sensor Arrays for Continuous Gas Leakage in Integrally Stiffened Plates Based on the Acoustic Characteristics of the Stiffener

**DOI:** 10.3390/s150924644

**Published:** 2015-09-23

**Authors:** Xu Bian, Yibo Li, Hao Feng, Jiaqiang Wang, Lei Qi, Shijiu Jin

**Affiliations:** 1 State Key Laboratory of Precision Measurement Technology and Instrument, Tianjin University, Tianjin 300072, China; E-Mails: bx332@tju.edu.cn (X.B.); fenghao@tju.edu.cn (H.F.); wangjiaqiang@tju.edu.cn (J.W.); shjjin@tju.edu.cn (S.J.); 2 Vacuum and Leak Detecting Division Beijing Institute of Spacecraft Environment Engineering, NO. 104 Youyi Road, Haidian District, Beijing 100094, China; E-Mail: qltjdx@aliyun.com

**Keywords:** continuous ultrasound, gas leakage, location, stiffener, sensor array

## Abstract

This paper proposes a continuous leakage location method based on the ultrasonic array sensor, which is specific to continuous gas leakage in a pressure container with an integral stiffener. This method collects the ultrasonic signals generated from the leakage hole through the piezoelectric ultrasonic sensor array, and analyzes the space-time correlation of every collected signal in the array. Meanwhile, it combines with the method of frequency compensation and superposition in time domain (SITD), based on the acoustic characteristics of the stiffener, to obtain a high-accuracy location result on the stiffener wall. According to the experimental results, the method successfully solves the orientation problem concerning continuous ultrasonic signals generated from leakage sources, and acquires high accuracy location information on the leakage source using a combination of multiple sets of orienting results. The mean value of location absolute error is 13.51 mm on the one-square-meter plate with an integral stiffener (4 mm width; 20 mm height; 197 mm spacing), and the maximum location absolute error is generally within a ±25 mm interval.

## 1. Introduction

For some special application requirements, such as spacecraft, stiffeners are used to add structural rigidity with minimal additional weight. In addition, the requirements of safety protection for this kind of vessel are higher. Once any position on the wall of the vessel is hit or corroded so that a hole is formed, leakage occurs, and it can affect the tightness of vacuum structures, reduce the system operational safety coefficient, and cause economic losses. Thus an effective detection method which can quickly identify the leakage source is very necessary.

According to different theories, current leakage detection technology mainly employs four methods: optical methods [1,2,3], the pressure change method [4], the resistance change method [5] and the acoustic emission (AE) location method. The AE location method involves analyzing the signal collected by the AE sensors at different positions to obtain the time difference information and then get the leakage source location through calculation (the typical one is the time difference of arrival (TDOA) technique [6]). Among these four methods, the AE location method is easy to implement; the structure of the detected object does not need to be changed, and it has a high location speed and high immunity to interference. However, the signal generated by the existing leakage is continuous ultrasonic broadband noise without time domain features, and the propagation characteristics are complicated [7,8,9,10,11] with regard to obtaining the information in the time domain. Thus the position cannot be determined using the traditional AE location method [6]. Therefore, the traditional AE location method presents some shortcomings in locating the continuous leakage source and it needs to be further explored. Currently, there has less reference about continuous gas leakage location specific to a vessel with a stiffener based on the acoustic method. Meng *et al*. [12] performed an acoustic experimental study on leak detection and localization for gas pipelines, conducted on a high-pressure and long-distance leak test loop. The researchers found that most acoustic leak signals were within the 0–100 Hz range, and they used different de-noising methods for different noise signals to improve the leakage location formula considering the pressure and temperature. Kitajima *et al*. [13] determined the leakage source position by considering the fact that the AE signal attenuates with the distance. However, this method is easily affected by the structure of the detected object and background noise, so it has a large location error under normal circumstances. Some ultrasonic leak detection equipment like UL101 [14,15] is used to locate leakages. The equipment locates the leakage source by collecting the ultrasonic leakage signal from the air surrounding the leakage holes. However, its detection range is small, and the equipment needs to be used to manually scan in every suspicious area, so the method is time-consuming. An *et al*. [16] have analyzed in detail the propagation rules about ultrasonic in a stiffener, and proposed a novel Lamb wave line-sensing technique for crack detection in a welded stiffener. Yelve *et al*. [17] have done similar research; they presented a study which focused on detection and quantification of the disband present beneath the stiffener in a stiffened aluminum panel, using a Lamb-wave-based nonlinear technique. Reusser *et al*. analyzed the acoustic characteristics of a stiffener in the frequency domain. Based on a specific frequency band, they proposed a sensor array to collect ultrasonic signals of orbiting spacecraft leaks [18,19,20,21] and calculated the intensity distribution of the wave number diagram (k-domain) of the collected signal in the specific frequency band, to estimate the direction of the sound source. Meanwhile, two sets of array orientation results are used to locate the leakage. However, this method requires a large number of sensors in the array (at least 64), and the location accuracy is poor (the biggest location error is 20 mm in a one square meter plate). Besides, this method needs the mechanical rotation array sensor when measuring, so it is difficult to implement. A method has been previously proposed by us [22] which uses sensor arrays for gas leakage location based on correlation of the time-space domain of continuous ultrasound. It obtains a location for the leakage holes on the container wall without stiffener, and its maximum location error is generally within a ±10 mm interval. However, the acoustic condition of the stiffener is more complicated, so the current method normally cannot get a correct location result, and it needs to be further analyzed and researched.

Specific to the vessel with the integral stiffener, this paper analyzes the characteristics of ultrasonic propagation across the stiffener, and proposes a new method for the location of continuous leakage sound sources. Experimental results indicate that this algorithm can locate leakage sources accurately. Compared with the previous location method [18,19,20,21], it can achieve similar location accuracy with a smaller number of sensors in one array, which means there is less total data to analyze, so the requirements on the data analysis system are reduced. Additionally, it does not need to make a special circuit for collection, so the structure of the location system is simpler and easier to apply.

## 2. Location Method

**Figure 1:**
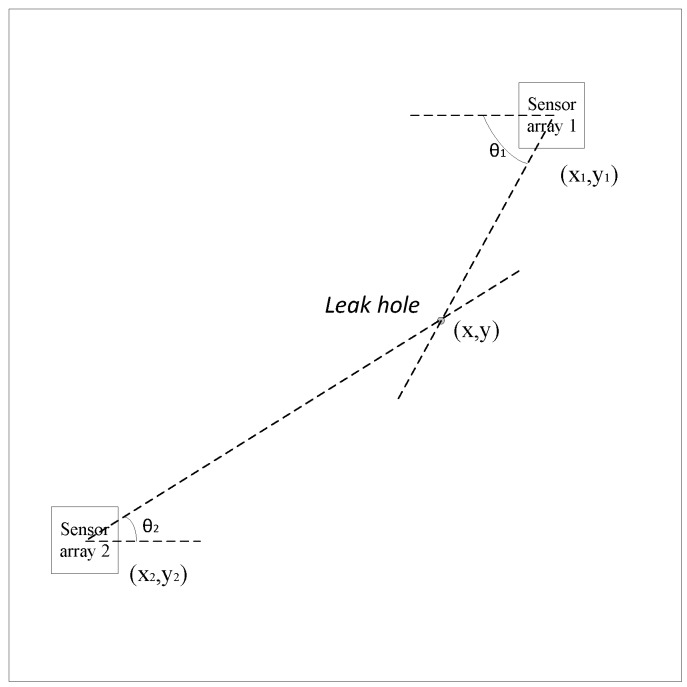
Locating principle diagram.

In practice, the ultrasonic signal generated by the same leakage source is continuous and stable, and the leakage location can be obtained by combining multiple sets of results from sensor arrays oriented in different positions, as shown in Figure 1.

In Equation (1), the parameters (*x_1_*,*y_1_*), (*x_2_*,*y_2_*) are known, thus to obtain the source position (x,y), θ_1_ and θ_2_ are necessary:

(1) $$\{\begin{array}{c} y-y_{1}=\tan(2\pi -\theta_{1})\;(x-x_{1})\\ y-y_{2}=\tan(\pi +\theta_{2})\;(x-x_{2}) \end{array}$$

**Figure 2:**
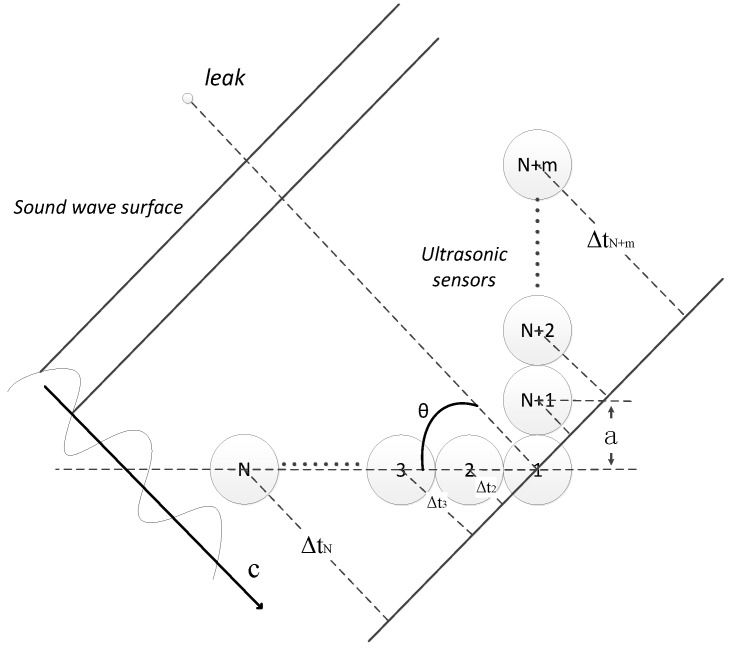
The model of the sensor array.

According to Equation (1), the location problem can be seen as a directional problem. However, it is difficult to solve the directional problem using a continuous signal by the traditional method, so how to obtain a high accuracy value for θ_1_ and θ_2_ has become the key issue. The model of the sensor array we have used is shown in Figure 2. In practical location, this type of array can obtain a high directional accuracy at the least system overhead. The process of analysis and testing the variable array directional characteristics is widely discussed in [23,24,25].

*N* + *m* represents the total amount of sensors in the array, where N and m are the number of sensors in the horizontal and vertical directions, respectively; *a* represents the center-to-center distance between two equally-spaced sensors; *c* is the sound velocity; *θ* represents the angle between the leak direction and the reference direction.

Supposing $p_{i}(t)\;(i\;=\;1,\;2,\;\ldots \;N\;+\;m)$ is the ultrasound signal collected by the *i*-th sensor, and let:

(2) $$P(t)=\left[\begin{array}{c} p_{1}(t)\\ p_{2}(t)\\ \vdots \\ p_{N+m}(t) \end{array}\right]$$

According to the geometrical relationship, at a specific angle *θ* and the speed *c*, the signal from the *i*-th sensor has a certain arrival time difference $\text{Δ}t_{i}(\theta )$, compared to the reference sensor. In this research, the first sensor is defined as the reference sensor.

(3) $$\begin{array}{l} \text{Δ}t_{i}(\theta )=\exp[2j\pi f\cdot \frac{a}{c}\Psi_{i}(\theta )]\\ \Psi_{i}(\theta )=\{\begin{array}{l} \begin{array}{cc} i\cdot \cos\theta & i=1,2,\cdots ,N \end{array}\\ \begin{array}{cc} (i-N)\cdot \sin\theta & i=N+1,N+2,\cdots N+m \end{array} \end{array} \end{array}$$

Due to the fact that the leakage signal is broadband, the frequency dispersion phenomenon exists when that signal propagates in the thin plate [26]. Thus, the sound velocity *c* depends on the frequency *f* which continuously changes. According to the previous research [23], the A0 mode has a greater contribution to the locating result than the other modes under the conditions we considered (the plate is less than 6 mm thick, and the signal frequency within the range 100–300 kHz). Thus, only the A_0_ mode has to be considered, and the *c* can be written as $c_{A_{0}}(f)$, and Equation (3) can be written as:

(4) $$\text{Δ}t_{i}(\theta ,f)=\exp[2j\pi f\cdot \frac{a}{c_{A_{0}}(f)}\Psi_{i}(\theta )]$$

Meanwhile, the $\text{Δ}t_{i}(\theta ,f)$ can be rewritten in matrix form:

(5) $$T(\theta ,f)=\left[\begin{array}{cccc} \text{Δ}t_{1}(\theta ,f), & \text{Δ}t_{2}(\theta ,f), & \cdots & ,\text{Δ}t_{N+m}(\theta ,f) \end{array}\right]$$

Selecting the time window as (t_a_,t_b_) and integrating Equation (2) and Equation (5), the energy output of sensor array (*E*) can be obtained under the specific angle θ:

(6) $$E_{t_{a}-t_{b}}(\theta ,f)=\int_{t_{a}}^{t_{b}}Τ(\theta ,f)P(t,f)dt$$

It can be proved by rigorous mathematical deduction that $E_{t_{a}-t_{b}}(\theta ,f)$ will get the maximum value when θ is same as the direction of arrival, and the deduction process has been described in the reference [23]. However, the stiffener acts as a geometry-dependent filter of the guided wave, so it has different influences on each frequency band signal. It is observed that certain frequency bands have high transmission and other frequency bands have high reflection [27]. Thus, the $E_{t_{a}-t_{b}}(\theta ,f)$ that was calculated by the band-width signals which we considered needs to be modified to make sure θ is the same as the direction of arrival. For analysis, the model of the integral stiffener can be described as below:

**Figure 3:**
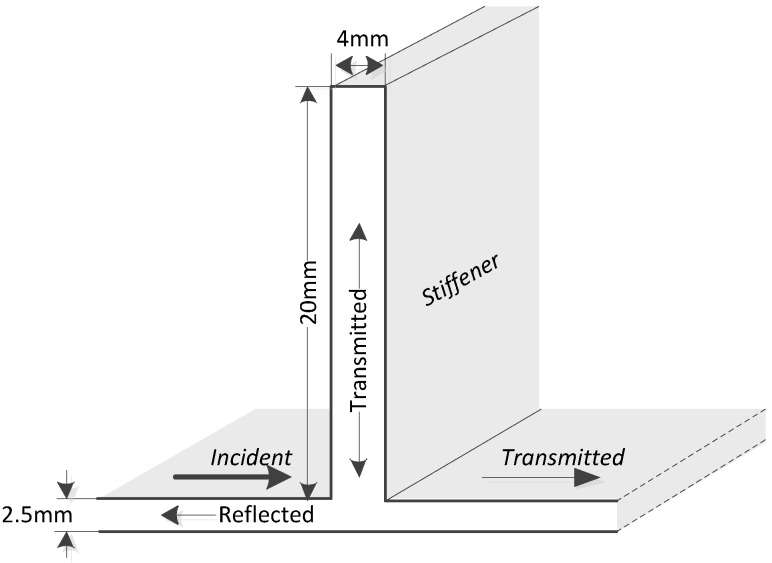
The model of ultrasonic propagation in an integral stiffener.

According to the Figure 3, the process of ultrasonic transmitting across the stiffener can be simplified as below:

(7) $$S_{I}(t)=S_{R}(t)+S_{Ts}(t)+S_{Tp}(t)$$

$S_{I}(t)$ represents the incident wave, $S_{R}(t)$ represents the reflected wave $S_{Ts}(t)$ represents the transmitted wave in the stiffener, and $S_{Tp}(t)$ represents the transmitted wave in the plate (behind the stiffener). By using the laser vibrometer, the vibration of a particle at any position on the surface of the plate and the stiffener could be obtained (the detailed method of the experiment is described in Section 3.1). Through analysis of the collected data, it is found that the power of the wave $S_{Ts}(t)$ is lower, and it could transform into $S_{R}(t)$ and finally $S_{Tp}(t)$. Thus, it has less influence for the ultrasonic signal transmission, and it can be ignored. In addition, a single incident mode will create many different scattered modes. The overall behavior is represented with an energy transmission coefficient; thus, when frequency is *f*, let $E_{I}(f),E_{R}(f),E_{T_{P}}(f)$ represent the energy of the incident wave, reflected wave and transmitted wave in the plate respectively. Due to the law of conservation of energy:

(8) $$E_{I}(f)=E_{R}(f)+E_{T_{P}}(f)$$

Let $\alpha =\frac{E_{T}(f)}{E_{R}(f)+E_{I}(f)}$, then the energy transmission coefficient can be defined as $H(f)=\frac{E_{T}(f)}{E_{I}(f)}=\frac{2\alpha }{1+\alpha }$. Thus Equation (6) can be rewritten as:

(9) $$E_{t_{a}-t_{b}}(\theta ,f)=\int_{t_{a}}^{t_{b}}T(\theta ,f)P(t,f)\cdot H(f)dt$$

Due to Equation (7), there is an obvious reflection for the ultrasonic when it goes across the stiffener. Thus, the collected signals from each sensor of the array are the result of superposition by the incident signal $p_{I}(t)$ and n times reflected signals $p_{R_{i}}(t)\;(i=1,2,\cdots n)$. They can be put into P(t) to obtain the following:

(10) $$P(t)=\left[\begin{array}{c} p_{1,I}(t)+p_{1,R_{1}}(t)+\cdots +p_{1,R_{n}}(t)\\ p_{2,I}(t)+p_{2,R_{1}}(t)+\cdots +p_{2,R_{n}}(t)\\ \vdots \\ p_{N+m,I}(t)+p_{N+m,R_{1}}(t)+\cdots +p_{N+m,R_{n}}(t) \end{array}\right]$$

According to the matrix algorithm, P(t) can be written as:

(11) $$P(t)=\left[\begin{array}{c} p_{1,I}(t)\\ p_{2,I}(t)\\ \vdots \\ p_{N+m,I}(t) \end{array}\right]+\left[\begin{array}{c} p_{1,R_{1}}(t)\\ p_{2,R_{2}}(t)\\ \vdots \\ p_{N+m,R_{n}}(t) \end{array}\right]+\cdots +\left[\begin{array}{c} p_{1,R_{n}}(t)\\ p_{1,R_{n}}(t)\\ \vdots \\ p_{N+m,R_{n}}(t) \end{array}\right]$$

Thus, under the frequency band of $\left(f_{c},f_{d}\right)$, Equation (9) becomes:

(12) $$\begin{array}{l} E_{t_{a}-t_{b}}(\theta )=\int_{t_{a}}^{t_{b}}\int_{f_{c}}^{f_{d}}T(\theta ,f)P(t,f)\cdot H(f)dfdt\\ =\int_{t_{a}}^{t_{b}}\int_{f_{c}}^{f_{d}}H(f)\cdot T(\theta ,f)\left\{\left[\begin{array}{c} p_{1,I}(t,f)\\ p_{2,I}(t,f)\\ \vdots \\ p_{N+m,I}(t,f) \end{array}\right]+\left[\begin{array}{c} p_{1,R_{1}}(t,f)\\ p_{2,R_{1}}(t,f)\\ \vdots \\ p_{N+m,R_{1}}(t,f) \end{array}\right]+\cdots +\left[\begin{array}{c} p_{1,R_{n}}(t,f)\\ p_{1,R_{n}}(t,f)\\ \vdots \\ p_{N+m,R_{n}}(t,f) \end{array}\right]\right\}dfdt\\ =E_{I,t_{a}-t_{b}}(\theta )+E_{R_{1},t_{a}-t_{b}}(\theta )+\cdots +E_{R_{n},t_{a}-t_{b}}(\theta )\\ =E_{I,t_{a}-t_{b}}(\theta )+\sum_{i=1}^{n}E_{R_{i},t_{a}-t_{b}}(\theta ) \end{array}$$

In practice, $E_{I,t_{a}-t_{b}}(\theta )>E_{R_{i},t_{a}-t_{b}}(\theta )$. Let φ represents the direction of leakage; when the *n* (the number of times the wave is reflected) is lower, there exists a θ=φ to make $E_{t_{a}-t_{b}}(\theta )$ become the maximum. However, the energy of the reflected signal could exceed the leakage signal, which means there exists a θ′≠φ to make $E_{t_{a}-t_{b}}(\theta \prime )$ become the maximum. The leakage signal is the noise whose amplitude and phase is random; meanwhile, the reflected signal generated by the noise in the stiffener is unstable, so the value of θ′ will change following the time window (t_a_,t_b_). While the leakage source continuously exists and is stable, $E_{I,t_{a}-t_{b}}(\varphi )$ exists stably compared with the reflected wave. The time window $\left(t_{0},t_{end}\right)$ we considered has been divided into L different fragments, and each E(θ) which is calculated from those fragments is superimposed to obtain $E_{t_{0}-t_{end}}(\theta )$ as below:

(13) $$\begin{array}{l} E_{t_{0}-t_{end}}(\theta )=E_{t_{0}-t_{j}}(\theta )+E_{t_{j+1}-t_{2j}}(\theta )+\cdots +E_{t_{(L-1)j+1}-t_{Lj}}(\theta )\\ =L\cdot E_{I,t_{1}-t_{j}}(\theta )+\sum_{i=1}^{n}E_{R_{i},t_{1}-t_{j}}(\theta )+\sum_{i=1}^{n}E_{R_{i},t_{j+1}-t_{2j}}(\theta )+\cdots +\sum_{i=1}^{n}E_{R_{i},t_{(L-1)j+1}-t_{Lj}}(\theta ) \end{array}$$

Let:

(14) $$E_{R}(\theta )=\sum_{i=1}^{n}E_{R_{i},t_{1}-t_{j}}(\theta )+\sum_{i=1}^{n}E_{R_{i},t_{j+1}-t_{2j}}(\theta )+\cdots +\sum_{i=1}^{n}E_{R_{i},t_{(L-1)j+1}-t_{Lj}}(\theta )$$

Thus Equation (14) can be written as:

(15) $$E_{t_{0}-t_{end}}(\theta )=L\cdot E_{I,t_{1}-t_{j}}(\theta )+E_{R}(\theta )$$

**Figure 4:**
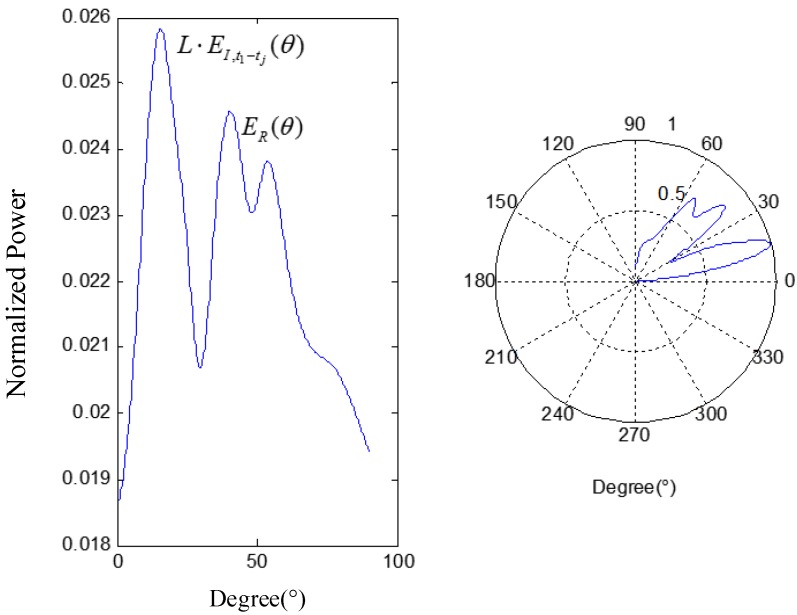
Angle-power relation.

According to Equation (15), the energy of the signal in the leakage direction will be enhanced, and the sum of reflected signals’ energy $E_{R}(\theta )$ will be decreased due to the randomness of each item in Equation (14), which means the contribution of $E_{R}(\theta )$ lessens in $E_{t_{0}-t_{end}}(\theta )$. Figure 4 plots the relationship between the angle *θ* and the normalized power *E*, calculated with numerical simulation using MATLAB^®^: the angle corresponding to the maximum power peak gives the estimated position of the acoustic source. 

**Figure 5:**
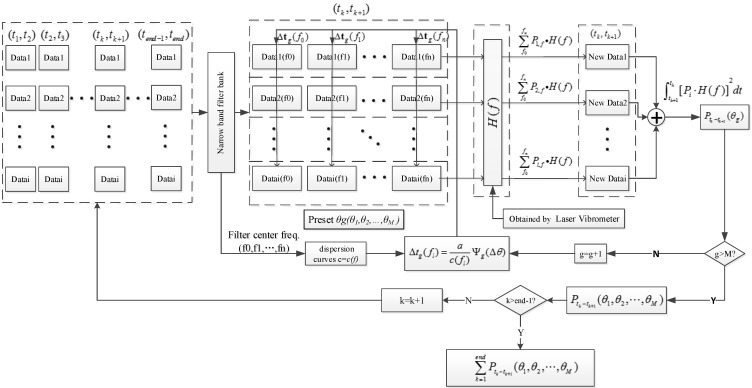
Algorithm flow chart.

The algorithm flow chart has been summarized in Figure 5. A narrow band filter has been used to obtain the required band signal with a frequency band that is so narrow that the velocity of sound can be regarded as a unique value.

## 3. Experimental

### 3.1. Research on the Parameters of H(f)

**Figure 6:**
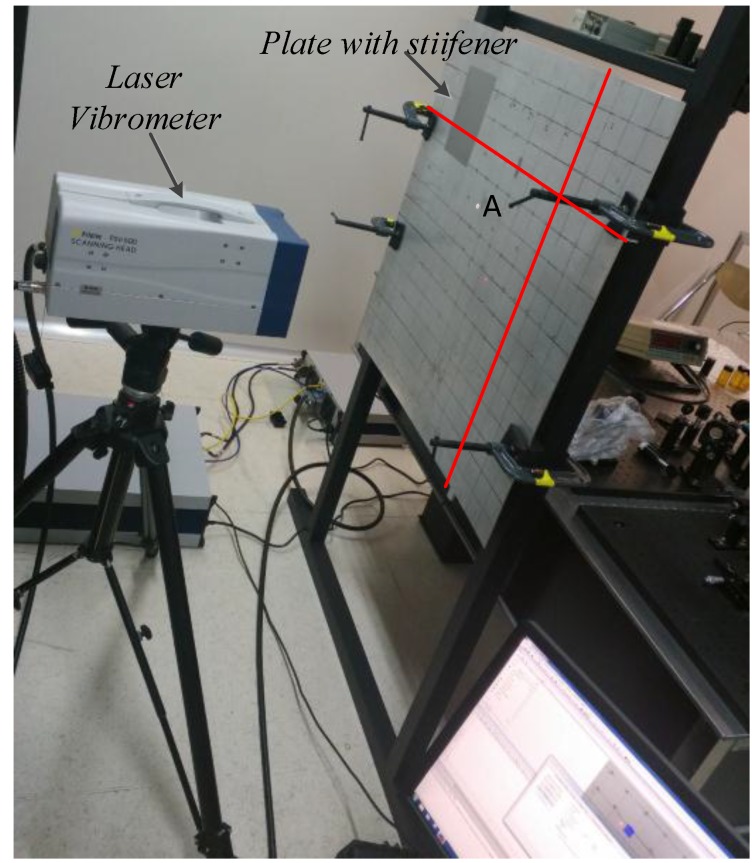
Experimental apparatus.

According to Equation (12), H(f) is the key parameter that needed to be obtained. Thus, an experiment was performed as described below to obtain H(f). A Polytec PSV-500 laser vibrometer has been used to measure the every movement of each surface particle on the plate to obtain the amplitudes of incident and transmitted guided waves, which were created by a piezoelectric ultrasonic transducer. Figure 6 shows the experimental apparatus. 

Experimental tests have been carried out using a magnesium aluminum alloy plate (the type is 5A06 which include two main elements: Mg accounts for 5.8%–6.8%, and Al accounts for more than 90%), which is an 800 × 800 × 2.5 mm plate with 20 mm × 4 mm integral stiffeners (as the red line show in Figure 6). The non-stiffener side was used in the experiment to ensure the measurement precision of the laser vibrometer. In the experiment, an ultrasonic transducer was used to generate the signal we need, and the ultrasonic generated signals could collected by the laser vibrometer. *A* represents the location of ultrasonic transducer and the gray part of the plate is the area scanned by the laser vibrometer. To obtain the best results, the full usable spans of the plates are scanned with 1 mm spacing. The schematic diagram of the experimental is shown below.

**Figure 7:**
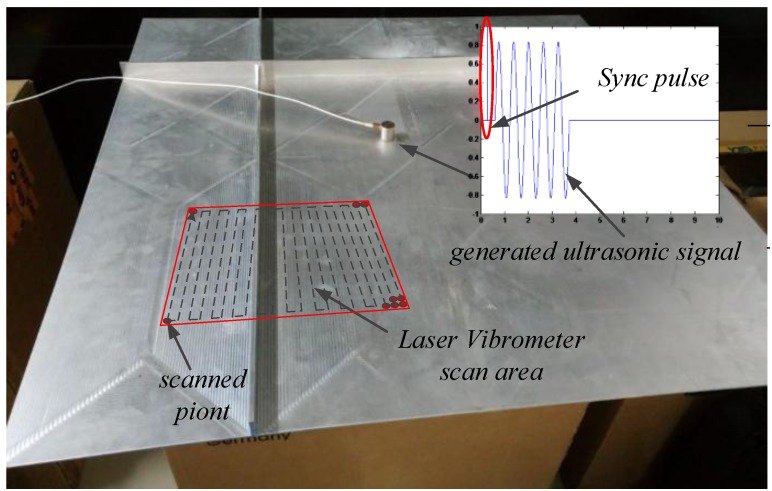
The schematic diagram.

The signal generator was used to generate the ultrasonic signals as needed, and simulate the sound source through the ultrasonic transducer. Meanwhile, the pulse signal (sync pulse) was added before the signal we generated to synchronize the clocks of the signal generator and the Polytec PSV-500 laser vibrometer to ensure the sending time and the collecting time of the signal are same. When the scanned point moves to the next one as shown in Figure 7, the same ultrasonic signal with sync pulse has been repeatedly sent. Analyzing the signal, which was collected by the acquisition system at every scanned point, the diagram of signal propagates in time domain can be obtained. Take the 200 kHz frequency as an example.

**Figure 8:**
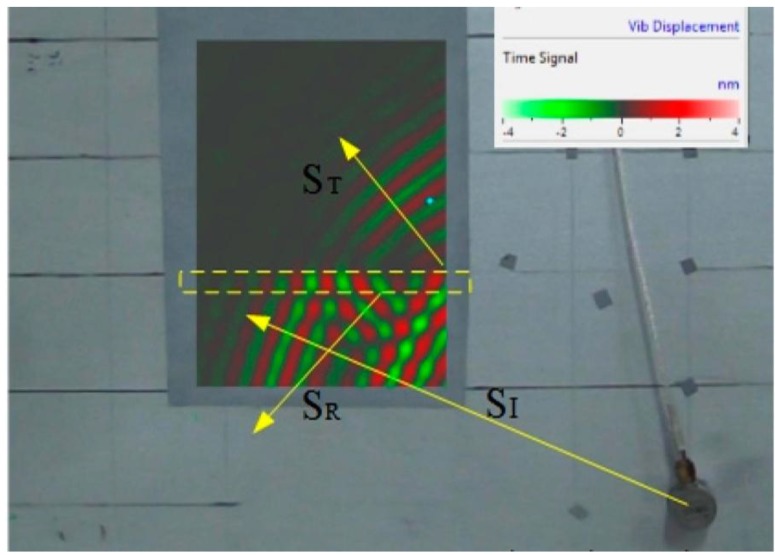
The signal propagate diagram (at 0.15392 ms).

The dotted box represents the integral stiffener position. According to Figure 8, there were fewer ultrasonic signals transmitted by the transmitted wave when they transmit across the stiffener, while large parts of signals were transmitted by the reflected wave, as analyzed in Section 2. Integrating the signal collected from each scanned point in the time domain, the energy space distribution diagram around the stiffener can be calculated as shown below.

**Figure 9:**
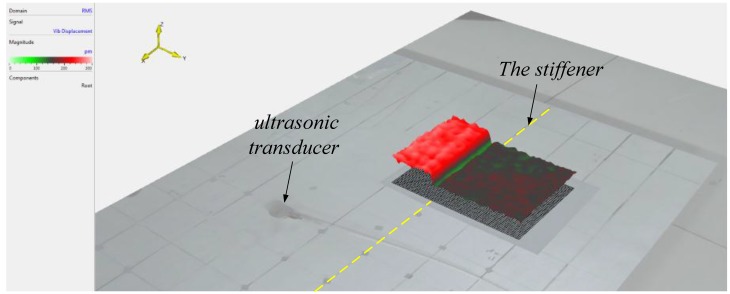
The energy space distribution diagram around the stiffener.

**Figure 10:**
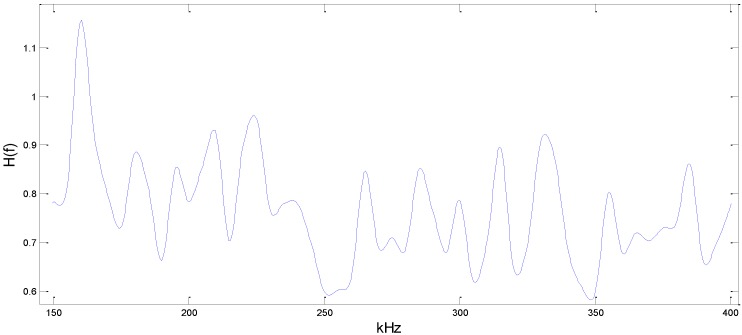
The transmission coefficients *H*(*f*).

According to Figure 9, the mean value of energy of the area we considered can be obtained so that the H(f) under frequency (*f*) is calculated. Moreover, different frequencies of ultrasonic signals are generated from the signal generator, and the curve H(f) about the transmission coefficients of different-frequency (*f*) signals’ energy in Equation (9) can be drawn by this method as shown in Figure 10.

### 3.2. The Experiment for Location

**Figure 11:**
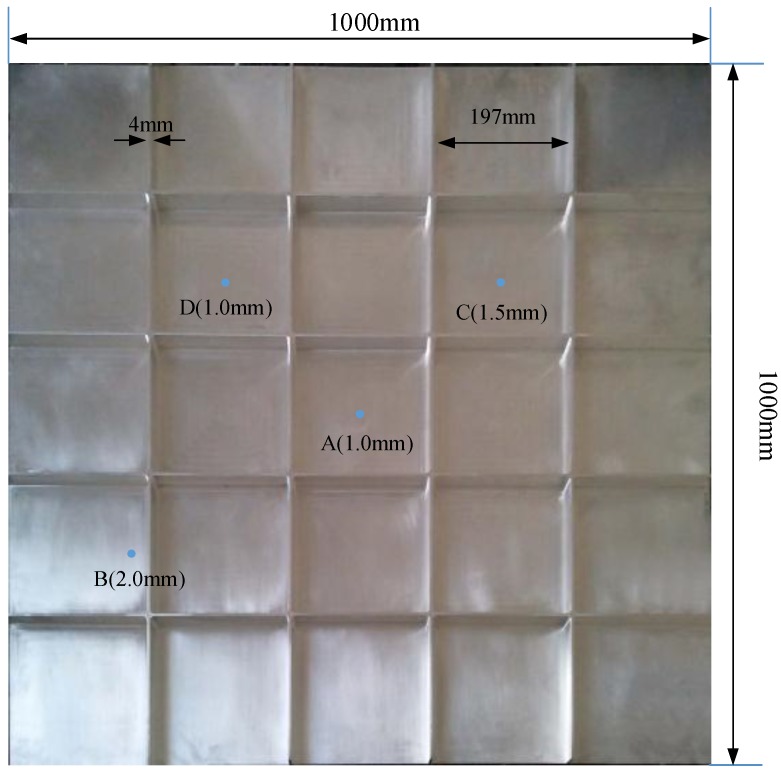
The test plate.

A magnesium aluminum alloy (the type is 5A06 which includes two main elements: Mg accounts for 5.8%–6.8%, and Al accounts for more than 90%) plate with integral stiffener has been machined to simulate the wall of a pressurized vessel with integral stiffener, as shown in Figure 11.

**Figure 12:**
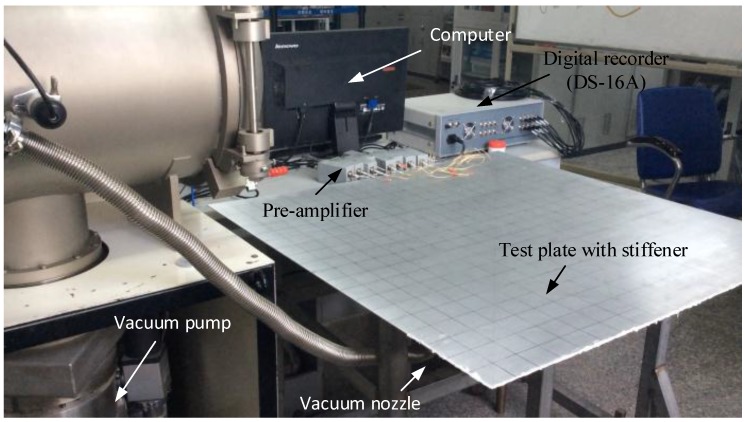
Experimental apparatus.

The plate is square (1000 × 1000 mm) and 2.5 mm thick. Referring to the requirements of the experiment, the width of stiffeners on the plate is 4 mm, the height is 20 mm, and the space between two stiffeners is 197 mm. A series of circular holes was drilled randomly on the surfaces to simulate leakage holes with different apertures; the size of the holes varied within the range 1–2 mm (in diameter). Some representative holes with the aperture are marked on Figure 11 for reference. A vacuum pump with a vacuum nozzle provided the loading, to maintain the leakage pressure. The leakage hole is connected with the vacuum pump through the vacuum suction nozzle. By starting the vacuum pump, air is drawn off from the vacuum nozzle and a continue leakage source is simulated. The ultrasonic signal generated by the leakage can be detected and acquired. By acquiring the ultrasonic at different positions with the sensor array, the orientation errors at any angle can be calculated, to verify the accuracy and stability of the method we proposed. Acoustic data from the sensor array was acquired using a fully digital 16-channel recorder (DS-16A), at a sampling rate of 3 MHz, and sent to a PC. Saved experimental data were processed with the MATLAB^®^ software. The pre-amplifiers (gain set to 40 dB) were installed between the sensor array and the data acquisition system, to boost the signal and reduce the effects of noise and interference. The experiment platform is shown in Figure 12. During the experiment, every experiment at any angle and position was carried out many times repeatedly (more than 5 times, generally), to make sure the experimental result’s accuracy and consistency.

**Figure 13:**
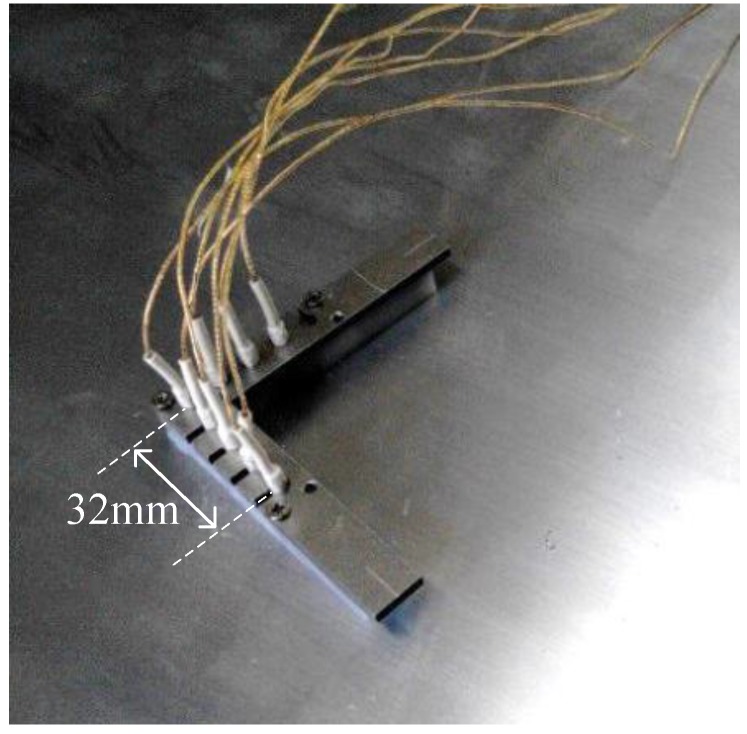
The 8-sensor array.

With reference to previous research [23], an L-type sensor array that is composed of eight AE sensors was selected. Nano-30 (Physical Acoustics Co, Princeton, NJ, USA) sensors were selected to ensure the best spatial response. The sensor array is shown in Figure 13; the center-to-center distance between two adjacent sensors is 8 mm.

## 4. Results and Discussion

**Figure 14:**
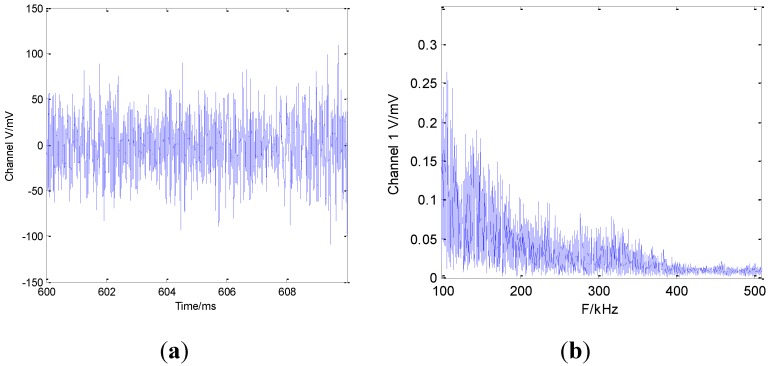
The time-frequency diagram of leakage signal. (**a**) Time domain diagram; (**b**) Frequency domain diagram.

The accuracy of this method has been validated through experimental tests described in this section. A series of experiments have been performed with the sensor array at a variety of locations on the test plate to verify the accuracy of the proposed method. Take one condition as an example: the actual direction of the leak is 38°, and the aperture of the leak is 1 mm. The time-frequency diagram of leakage signal collected by No.1 (as shown in Figure 2) sensor of the array is shown in Figure 14. 

For two different time windows *T_1_*, *T_2_* randomly, such as *T_1_*: 0.6 s–0.61 s, *T_2_*: 2 s–2.01 s for example, the orientation results are shown below. 

**Figure 15:**
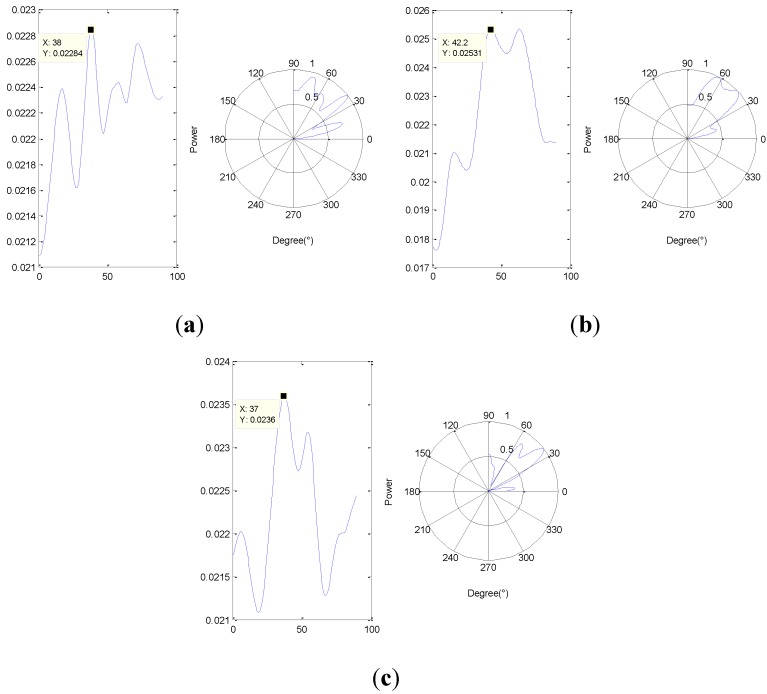
The orientation results (**a**) under *T_1_* and using *H(f)*; (**b**) under *T_1_* and not using *H(f)*; and (**c**) under *T_2_* and using *H(f)*.

According to Figure 15, there exist many peaks except the one which represents the leakage (fake-peak), and change follows the time window. Thus SITD can reduce the influence of those peaks on the leakage one in order to make sure orientation result is more stable. Comparing Figure 15a,b the distribution of fake-peak in the Figure 15a is more discrete, and the difference between the second maximum peak and the maximum peak is greater. Meanwhile, the orientation result is closer to the actual direction of the leak, so frequency compensation (using *H(f)*) can improve the orientation accuracy. To further show the influence on the orientation results from the two methods that mentioned above, several experimental data with three types of apertures (1 mm, 1.5 mm and 2 mm) were collected and analyzed. The orientation results without SITD in different time windows are unstable, so typical error is used to represent the most frequent among all of the results which were calculated in about 10 different time windows, and the value in the parenthesis indicates the second most frequent result, if it exists. The main experimental results are shown below.

**Figure 16:**
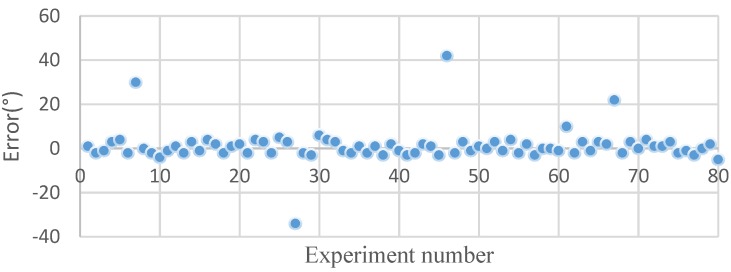
Orientation error.

According to Table 1, compared with the average absolute error 10.9 and variance 211, which are from the reference group without H(f) and SITD, the group with H(f) are at 3.8° and 97.0, respectively, the group with SITD are at 6.9° and 91.5, respectively. Meanwhile, the group with the two methods is at 2° and 1.7, respectively, which is better than others. More orientation results are shown in Figure 16.

**Table 1:** The orientation results comparison.

Aperture (mm)	No Using *H*(*f*)				Using *H*(*f*)			
	No Using SITD		SITD		No Using SITD		SITD	
	Typical Error (°)	Variance	Error (°)	Variance	Typical Error (°)	Variance	Error (°)	Variance
1.0	−12	62.5	−13	75.4	−10	10.2	−4	4.3
	−5 (42)	325.0	−3	37.0	−2 (40)	166.1	−1	1.5
	−4 (21)	206.2	3	23.8	−4	14.2	−1	2.2
1.5	−6 (31)	278.4	1	22.0	−3	13.4	−2	0.7
	−2 (30)	243.3	2	21.4	−2 (27)	104.2	−1	3.5
	−1 (22)	173.7	−1	82.2	−2	4.0	−2	0.7
2.0	−4	133.0	−2	4.2	−4	3.6	−2	1.3
	27 (3)	84.7	3	163.8	3 (27)	156.5	2	0.7
	37 (−3)	393.1	37	393.4	−4 (40)	400.9	−3	0.7
**Average Absolute Value**	10.9	211.1	6.9	91.5	3.8	97.0	2	1.7

**Figure 17:**
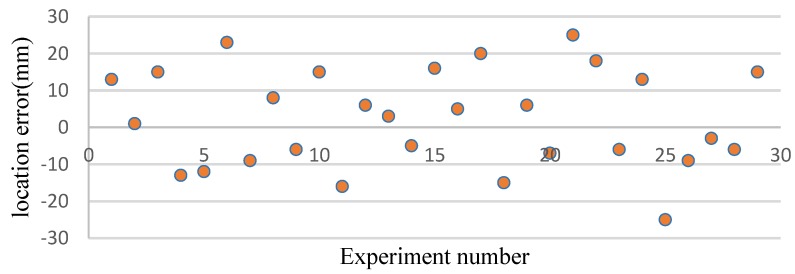
Location error.

In theory, if the orientation error is ≤±5°, the maximum location error will be less than 20 cm in the worst case from a geometric perspective (the sensors are maximally separated on the 1 m^2^ plate) and the probability is 92.5% obtained by the method we proposed. To verify the location performance of this method, two sensor arrays are placed at different positions randomly to detect the signal from any leakage hole. Some results are shown in Figure 17, and these results are typical of many others we have obtained.

The location error is defined as the distance between the estimated leakage position and the actual leakage hole. According to Figure 17, the maximum error of location is 25 mm, which is larger than one we obtained on the plate without a stiffener [22]. The mean absolute error is 16 mm, and the variance is 169.69. 

## 5. Conclusions

In order to solve the location problem of continuous gas leakage in pressurized vessels (such as spacecraft) with an integral stiffener, this paper proposes a location method which can obtain a high-accuracy location result on integrally stiffened plate. The conclusions have been summarized below.

1. The experiments showed that there exist obvious reflection waves and transmission waves when the ultrasonic propagates across the stiffener, and the ratio of those waves’ energy changes, followed by the frequency, as shown in Figure 10. By compensating for the frequency based on this phenomenon, we can increase the location accuracy to some extent.

2. According to the experiment results, the main location error is caused by the influence of the stiffener, and it is difficult to eliminate that influence by frequency compensation alone. Thus a method that combined frequency compensation and SITD has been proposed in this paper, which makes the accuracy and stability of the location results better to meet the location requirements. However, the acoustic characteristics of the stiffener are complicated, and the orientation results calculated by the method proposed in this paper still produce some results with large errors, as shown in Figure 15. Thus, a way to greatly improve the accuracy of orientation needs to be further researched. 
